# Practical Synthesis of Ethynyl(phenyl)-λ^3^-Iodane Using Calcium Carbide as an Ethynyl Group Source

**DOI:** 10.3389/fchem.2020.00012

**Published:** 2020-02-04

**Authors:** Takahiro Hashishin, Taisei Osawa, Kazunori Miyamoto, Masanobu Uchiyama

**Affiliations:** ^1^Graduate School of Pharmaceutical Sciences, The University of Tokyo, Tokyo, Japan; ^2^Research Initiative for Supra-Materials (RISM), Shinshu University, Ueda, Japan; ^3^Cluster of Pioneering Research (CPR), Advanced Elements Chemistry Laboratory, RIKEN, Saitama, Japan

**Keywords:** hypervalent, iodine, stannane, calcium carbide, ethynyl

## Abstract

Stannylation of calcium carbide followed by Sn–hypervalent iodine(III) exchange reaction cleanly afforded the electrophilic ethynylating agent ethynyl(phenyl)-λ^3^-iodane in high yield. This two-step method uses very inexpensive materials and is readily operable without any special precautions.

## Introduction

**Figure 1:**
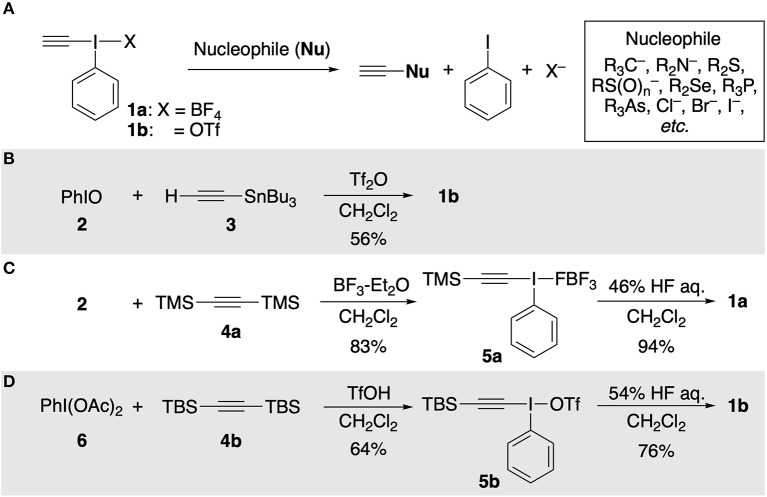
**(A)** Electrophilic ethynylation of nucleophile with **1**. **(B)** Stang's approach. **(C)** Ochiai's approach. **(D)** Kitamura's approach.

Hypervalent ethynyl(phenyl)-λ^3^-iodane **1** is an efficient electrophilic ethynylating agent for a variety of nucleophiles (*C, N, O, P, As, S, Se*, and halides) in the presence or absence of transition metal catalysts (Figure 1A; Ochiai et al., 1990; Stang et al., 1990; Varvoglis, 1992; Ochiai, 2003; Waser, 2016; Yoshimura and Zhdankin, 2016). However, its synthetic utility is restricted by its high cost and heat/moisture-sensitive character: it gradually decomposes at room temperature in air (Ochiai et al., 2003; Yudasaka et al., 2019). Therefore, an inexpensive, rapid, and facile preparation method of **1** has long been highly desired. The current approach to the synthesis of **1** relies on electrophilic Si/Sn–I(III) exchange reaction on acetylenic carbon atoms, and has remained essentially unchanged since the early days. In 1990, Stang and Ochiai independently reported pioneering approaches for the synthesis of **1**. Stang et al. prepared ethynyl(phenyl)(triflato)-λ^3^-iodane (**1b**) from ethynyl(tributyl)stannane (**3**) and Tf_2_O-activated iodosylbenzene (**2**) (Figure 1B; Stang et al., 1990). On the other hand, a two-step procedure for the synthesis of **1a** via [β*-*(trimethylsilyl)ethynyl](phenyl)-λ^3^-iodane **5a** was developed by Ochiai and co-workers (Figure 1C; Ochiai et al., 1990). Then, in 2011, Kitamura reported another practical stepwise approach using PhI(OAc)_2_ (**6**) and bis(*tert*-butyldimethylsilyl)acetylene (**4b**) (Figure 1D; Kitamura et al., 2011). Although these methods provide short-step approaches to **1**, they have several disadvantages from the viewpoint of cost/safety of reagents. In particular, **3** is still expensive and concentrated aqueous HF is notoriously toxic (Mckee et al., 2014). We report here a safe, low-cost, two-step method for the synthesis of **1a** using calcium carbide CaC_2_ (**7**) as an ethynyl group source.

Calcium carbide CaC_2_ (**7**) is a widely utilized industrial material that is very inexpensive (0.05 $/g; 3.2 $/mol) (Sigma-Aldrich Co., LLC.). However, its synthetic use has been limited by its poor solubility: it is not soluble in non-reactive common organic solvents (Barber and Sloan, 1961). In recent decades, several approaches using strongly coordinating solvents (DMSO, DMF, etc.) and/or coordinating additives (F^−^, ${\text{CO}}_{3}^{2-}$, HO^−^, water, etc.) have been reported, for which **7** served as a practical ${\text{C}}_{2}^{2-}$ source (Rodygin et al., 2016). It occurred to us that the treatment of **7** with inexpensive chloro(tributyl)stannane (**8**) (0.3 $/g; 98 $/mol) (Sigma-Aldrich Co., LLC.), followed by Sn–I(III) exchange reaction, might provide more convenient and straightforward access to **1**.

## Materials and Methods

Calcium carbide (~80%), ethynyl(tributyl)stannane (95%), bis(tributylstannyl)acetylene (95%) were purchased from Sigma Aldrich and used as received. Chloro(tributyl)stannane (>97%), and boron trifluoride etherate (>98%) were purchased from TCI Japan and used as received. (Diacetoxyiodo)benzene (98+%) was purchased from FUJIFILM Wako Pure Chemical or prepared according to the literature (Watanabe et al., 2018). Potassium carbonate (>99.5%) was purchased from FUJIFILM Wako Pure Chemical and used as received. Iodosylbenzene was prepared from (diacetoxyiodo)benzene according to the literature (Sharefkin and Saltzman, 1963). Anhydrous grade of dimethyl sulfoxide, tetrahydrofuran, and *N, N*-dimethylformamide was purchased from Kanto Chemical and degassed by purging with argon and/or dried with a solvent purification system containing a one-meter column of activated alumina.

### Characterization

NMR spectra were obtained on a Bruker AVANCE 500 spectrometer. Chemical shifts are expressed in δ (ppm) values. ^1^H NMR, ^13^C NMR, and ^19^F NMR spectra were referenced to tetramethylsilane (0 ppm), CHCl_3_ (7.26, 77.2 ppm), CHD_2_CN (1.94 ppm), and CD_3_CN (118.3 ppm), CFCl_3_ (0 ppm) as internal standards. IR spectra were obtained on a JASCO FT/IR-4700 spectrometer. Kieselgel 60 (Merck, 230-400 mesh) was used for column chromatography.

### Synthesis of Ethynyl(Tributyl)Stannanes

These reactions were carried out in a two-necked round bottom flask. In a typical reaction: To a stirred suspension of well-ground calcium carbide (**7**) (2.48 g, 38.7 mmol) in DMSO (20 mL) were added chloro(tributyl)stannane (**8**) (3.26 g, 10.0 mmol) and water (0.40 mL, 22.2 mmol) at room temperature under argon. The resulting grayish suspension was warmed to 80°C for 1 h (the disappearance of **8** was monitored by GCMS analysis), then allowed to cool to room temperature. Hexane was added to it, and the organic phase was filtered under reduced pressure through a K_2_CO_3_-silica gel (1:9) mixture and transferred to a separating funnel. The combined organic phase was washed with water several times, then filtered, and the filtrate was concentrated under reduced pressure to give an oil, which was further purified by chromatography (ø5 mm) on a column packed with K_2_CO_3_-silica gel (1:9). Elution with hexane gave a pale yellow oil (2.51 g). ^1^H NMR analysis (mesitylene as an internal standard) showed the formation of a mixture of ethynyl(tributyl)stannane (**3**) (3.4 mmol, 34%) and bis(tributylstannyl)acetylene (**9**) (2.3 mmol, 45%). Capillary GC analysis (n-dodecane as an internal standard; Bruker BR-5ms column 0.25 mm × 30 m, 100°C) showed different yields of **3** (3.6 mmol, 36%) and **9** (1.60 mmol, 32%), probably reflecting partial decomposition of **9** during the GC analysis. This product mixture was used directly for the synthesis of **1a**. Spectroscopic data of **3** and **9** were compared to the authentic samples synthesized according to the literatures (Supplementary Material).

Ethynyl(tributyl)stannane (**3**) (Stille and Simpson, 1987): a colorless oil; ^1^H NMR (CDCl_3_, 500 MHz) δ 2.20 (s, lH), 1.61–1.54 (m, 6H), 1.40–1.29 (m, 6H), 1.02 (t, *J* = 8.2 Hz, 6H), 0.91 (t, *J* = 7.3 Hz, 9H). ^13^C NMR (CDCl_3_, 125 MHz) δ 96.9, 89.1, 29.0, 27.1, 13.8, 11.2.

Bis(tributylstannyl)acetylene (**9**) (Brown and Eichler, 2011): a colorless oil; ^1^H NMR (CDCl_3_, 500 MHz) δ 1.69–1.48 (m, 12H), 1.40–1.29 (m, 12H), 1.13–0.94 (m, 12H), 0.90 (t, *J* = 7.3 Hz, 18H). ^13^C NMR (CDCl_3_, 125 MHz) δ 116.6, 29.1, 27.1, 13.8, 11.4.

The same procedure was adopted for other conditions shown in Table 1.

**Table 1:** Stannylation of CaC_2_ **7** with Bu_3_SnCl **8**.

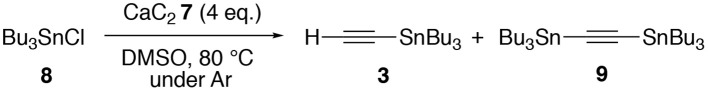				
**Entry**	**H**_**2**_**O** **(equiv)**	**Time** **(h)**	**Yield (%)**^**a**^	
			**3**	**9**
1	2	1	(20)	(43)
2^b^	2	1	(34) [36]^c^	(45) [32]^c^
3	2	6	(10)	(67)
4	2	28	0	72
5	0	28	0	65
6	12	3	[1]^c^	[50]^c^

*Reactions were carried out on 0.3–1 mmol scale*.

a *Isolated yields based on **8**, numbers in parentheses are ^1^H NMR yields*.

b *10 mmol scale*.

c *GC yields*.

### General Procedure for Synthesis of Ethynyl-λ^3^-Iodane 1a From PhI(OAc)_2_ 6

To a stirred solution of (diacetoxyiodo)benzene (**6**) (159 mg, 0.49 mmol) in dichloromethane (1 mL) was added BF_3_-Et_2_O (130 μL, 1.04 mmol) at −78°C, and then a 60:40 mixture of stannanes **3** and **9** (301 mg, 0.70 mmol) was slowly added. The reaction mixture was stirred at the same temperature for 1 h, then allowed to warm to room temperature, and the solvent was removed under reduced pressure. The resulting pale yellow solid was washed several times with hexane and Et_2_O at 0°C to give **1a** (114 mg, 73%).

Ethynyl(phenyl)(tetrafluoroborato)-λ^3^-iodane (**1a**) (Ochiai et al., 1990): a white solid; IR (ATR-FTIR) ν 3,241, 3,080, 2,056, 1,480, 1,442, 1,170–840, 735, 672 cm^−1^; ^1^H NMR (CD_3_CN, 500 MHz) δ 8.18 (d, *J* = 8.5 Hz, 2H), 7.82 (t, *J* = 7.6 Hz, 1H), 7.64 (dd, *J* = 8.5, 7.6 Hz, 2H), 3.89 (s, 1H). ^19^F NMR (CD_3_CN, 470 MHz) δ −151.8 (s, 4F). ^13^C NMR (CDCl_3_, 125 MHz) δ 136.1, 134.6, 133.8, 116.5, 99.0, 26.7 (see also Supplementary Material).

### General Procedure for Synthesis of Ethynyl-λ^3^-Iodane 1a From PhIO 2

To a stirred solution of iodosylbenzene (**2**) (77.6 mg, 0.35 mmol) in dichloromethane (0.7 mL) was added BF_3_-Et_2_O (100 μL, 0.77 mmol) at −78°C, and then a 38:62 mixture of stannanes **3** and **9** (218 mg, 0.46 mmol) was slowly added. The reaction mixture was stirred at the same temperature for 1 h, then allowed to warm to room temperature, and the solvent was removed under reduced pressure. The resulting pale brown solid was washed several times with hexane and Et_2_O at 0°C to give **1a** (90.4 mg, 81%); ^1^H NMR analysis shows this product contained a small amount of impurities. ^1^H NMR yield: 67% (mesitylene as an internal standard).

## Results and Discussion

We commenced our study by trapping CaC_2_ **7** with **8**. Exposure of well-ground **7** (4 equiv) to **8** in DMSO at room temperature did not give any alkynylstannanes. Addition of small amount of water (2 equiv), which has been reported to be effective for the electrophilic trapping of **7**, was fruitless (Rodygin et al., 2016). On the other hand, heating at 80°C resulted in smooth consumption of **8** and after 1 h, a 6:4 mixture of **3** and bis(tributylstannyl)acetylene (**9**) was obtained in 79% yield (Table 1, entry 2). The ratio of **3** and **9** has changed in a range of ca. 3:7–6:4 through several runs, partly due to the reaction scale and the surface area of **7** (entries 1 and 2). Use of longer reaction time increased the ratio of **9** (entries 3 and 4). Under the conditions, the addition of water did not significantly change the yield of **9**, but it accelerated the bis-stannylation (entries 4–6). Interestingly, this stannylation did not occur in other aprotic solvents such as THF and DMF, even at elevated temperatures (≤110°C) (Cochran et al., 1990). It should be noted that these alkynylstannanes **3** and **9** could be separated from other organostannane impurities on a short column packed with K_2_CO_3_-silica gel (1:9) mixture (Harrowven et al., 2010). Other crystallogen analog, trimethylsilyl chloride did not afford corresponding ethynyl(trimethyl)silanes under optimized conditions, partly because of the more moisture sensitive character of silyl chloride.

**Figure 2:**
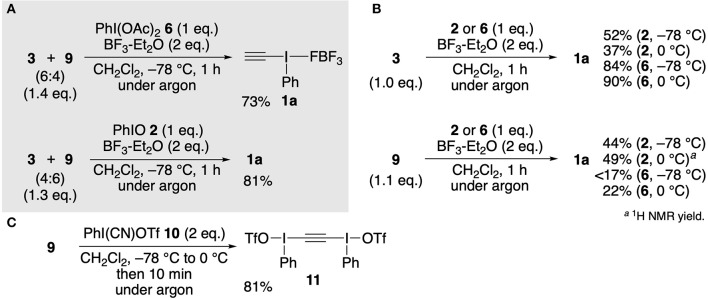
Synthesis of ethynyl(phenyl)-λ^3^-iodane **1a**. **(A)** Reaction of a mixture of alkynylstannanes **3** and **9** obtained from CaC_2_ **7**. **(B)** Individual reactions of authentic **3** and **9**. **(C)** Stang and Zhdankin's approach.

Next, we focused on the synthesis of ethynyl-λ^3^-iodane **1a** using a mixture of alkynylstannanes **3** and **9**. After screening various reaction conditions, we found an efficient method. Exposure of a 6:4 mixture of **3** and **9** (obtained from the reaction shown in entry 1 in Table 1) to a combination of PhI(OAc)_2_ **6** and BF_3_-Et_2_O in dichloromethane at −78°C resulted in smooth Sn–I(III) exchange, and after 1 h, **1a** was selectively obtained in 73% yield (Figure 2A). The standard PhIO **2**–BF_3_-Et_2_O system also afforded **1a** in high yield. It should be emphasized that these methods do not require time-consuming work-up. Simple washing of the reaction mixture with hexane and Et_2_O by decantation gave pure **1a** and a mixture of Bu_3_SnX-type organostannanes thus formed by I(III)–Sn exchange was recovered quantitatively in the supernatant. As we expected, these optimized conditions could also be applied to authentic **3** and **9** individually to provide **1a** in moderate to high yields (Figure 2B). In these cases, the combination of I(III)–organostannane pairs (**6**–**3** and **2**–**9**) gave better yields of **1a** than opposite pairs (**6**–**9** and **2**–**3**), although the reason remains unclear. From a mechanistic point of view, our results using **9** is somewhat surprising since the Sn–I(III) exchange of **9** with cyano(trifluoromethylsulfonyloxy)iodobenzene (**10**) selectively affords bis[phenyl(triflato)-λ^3^-iodanyl]acetylene (**11**) (Figure 2C; Stang and Zhdankin, 1990, 1991). The moderately electrophilic nature of iodine center of the intermediates such as PhI(OAc)_2_-BF_3_ (Izquierdo et al., 2016) or PhIO-BF_3_ (Ochiai, 2007) might be partly responsible for the selective formation of **1a**.

## Conclusion

In summary, we have developed a safe and inexpensive two-step method for the synthesis of **1a** using readily available CaC_2_ **7** as an ethynyl group source. This method not only provides time-/cost-/labor-saving methodology to prepare unstable ethynyl-λ^3^-iodane **1**, but also serves as an effective approach for synthetically useful but costly bis(stannyl)acetylene **9** (Brend'amour et al., 2018).

## Data Availability Statement

All datasets generated for this study are included in the article/Supplementary Material.

## Author Contributions

KM and MU conceived and designed the experiments and wrote the manuscript. TH and TO conducted the experiments. All authors participated in data analyses and discussions.

### Conflict of Interest

The authors declare that the research was conducted in the absence of any commercial or financial relationships that could be construed as a potential conflict of interest.
