# Supplementary material for: Practical Synthesis of Ethynyl(phenyl)-λ3-Iodane Using Calcium Carbide as an Ethynyl Group Source
Source: Front Chem. 2020 Feb 4;8:12. doi: 10.3389/fchem.2020.00012 (PMC7010717; doi:10.3389/fchem.2020.00012)
Supplement: Supplementary file 1 [file Data_Sheet_1.docx]

Supplementary Material

Practical synthesis of ethynyl(phenyl)-λ^3^-iodane using calcium carbide as an ethynyl group source

Takahiro Hashishin,^1^ Taisei Osawa,^1^ Kazunori Miyamoto,^1,^* Masanobu Uchiyama^1,2,3^*

^1^Graduate School of Pharmaceutical Sciences, The University of Tokyo, 7-3-1 Hongo, Bunkyo-ku, Tokyo 113-0033, Japan.

^2^Research Initiative for Supra-Materials (RISM), Shinshu University, Ueda, 386-8567, Japan.

^3^Cluster of Pioneering Research (CPR), Advanced Elements Chemistry Laboratory, RIKEN, 2-1 Hirosawa, Wako-shi, Saitama 351-0198, Japan

**Correspondence**: Kazunori Miyamoto, kmiya@mol.f.u-tokyo.ac.jp

**Table of Contents**

1. ^1^H and ^13^C NMR spectra S2-S4

^1^H and ^13^C NMR spectra of products **3**, **9**, and **1a**.


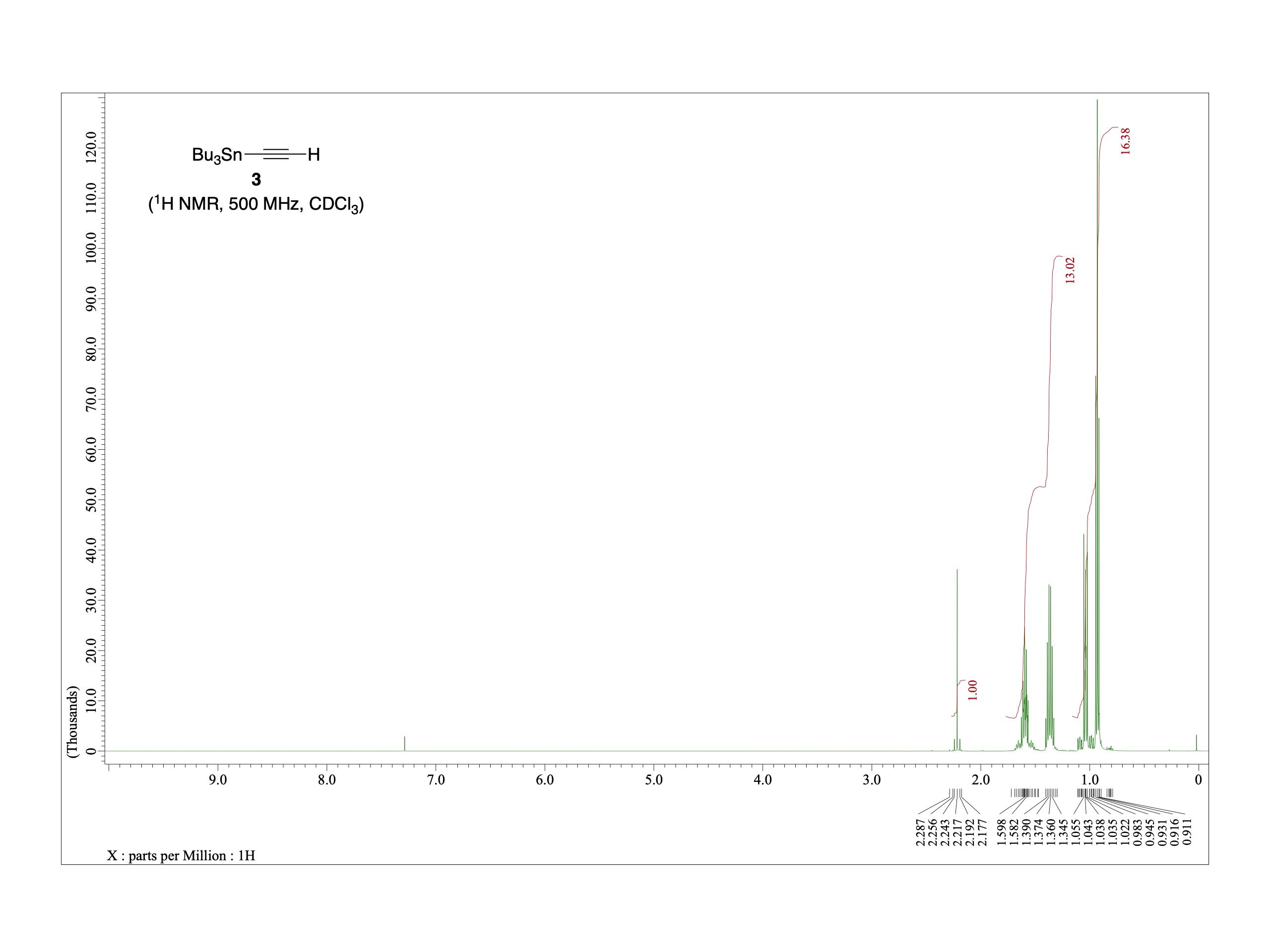


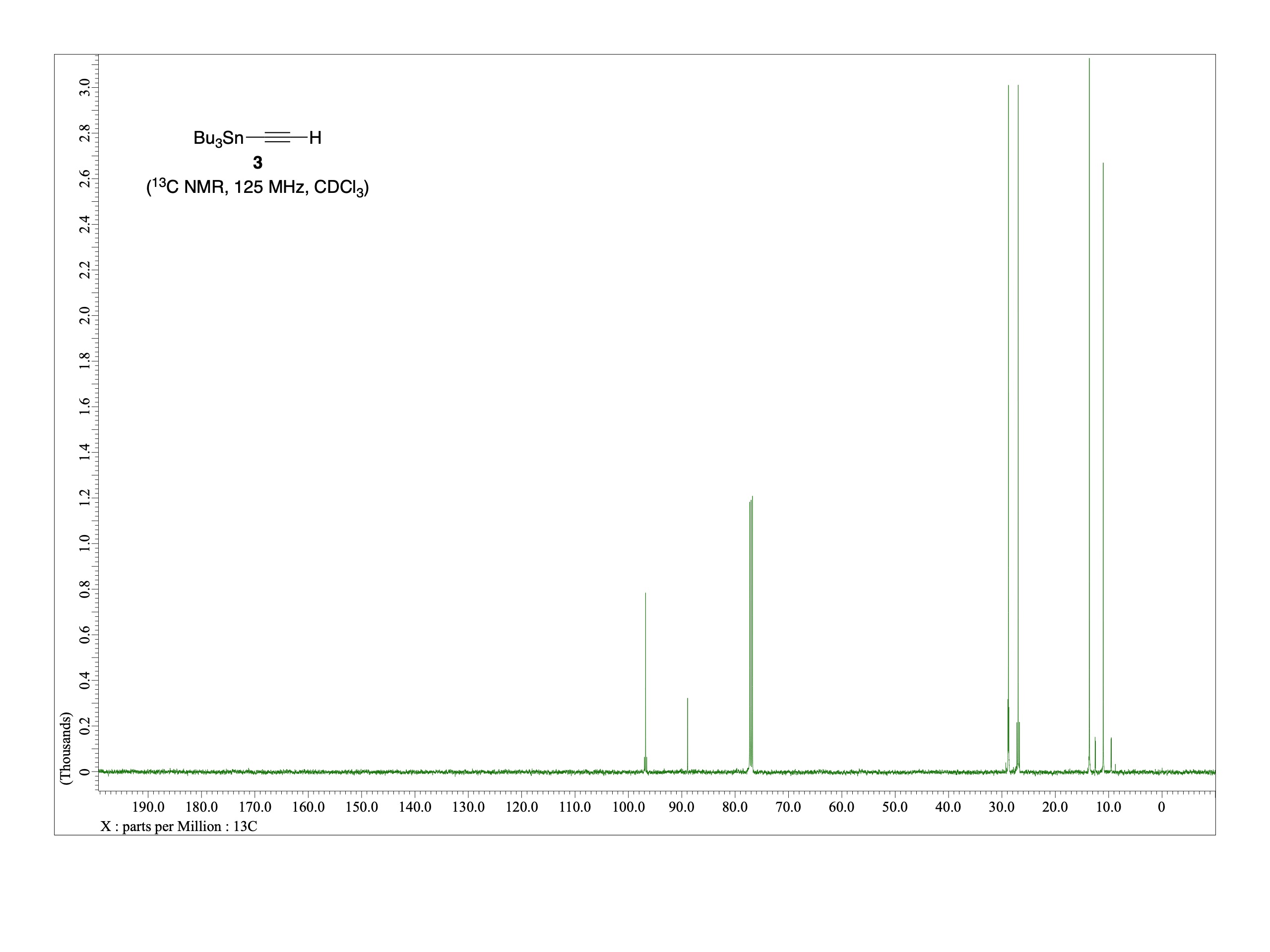


S2


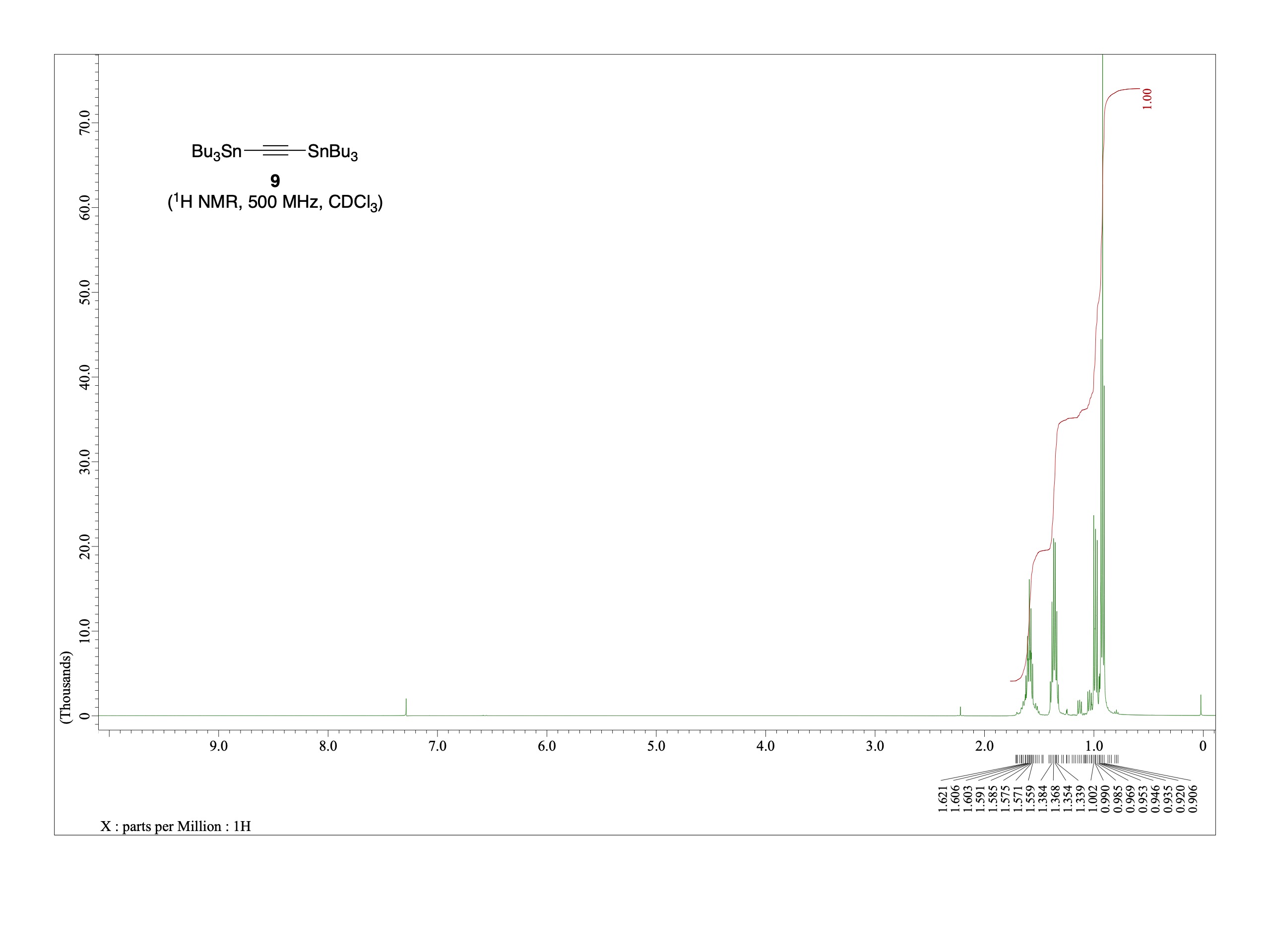

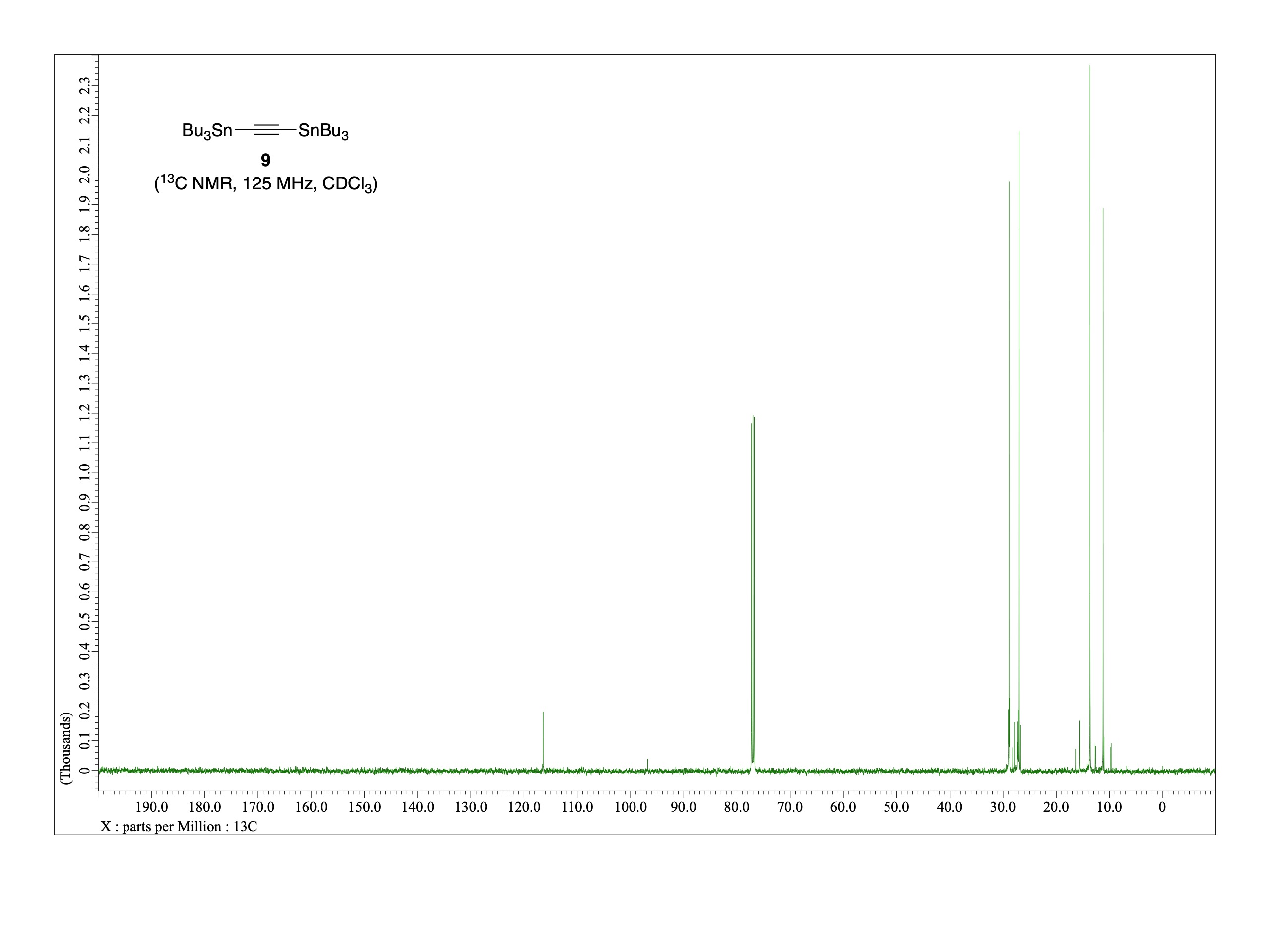


S3


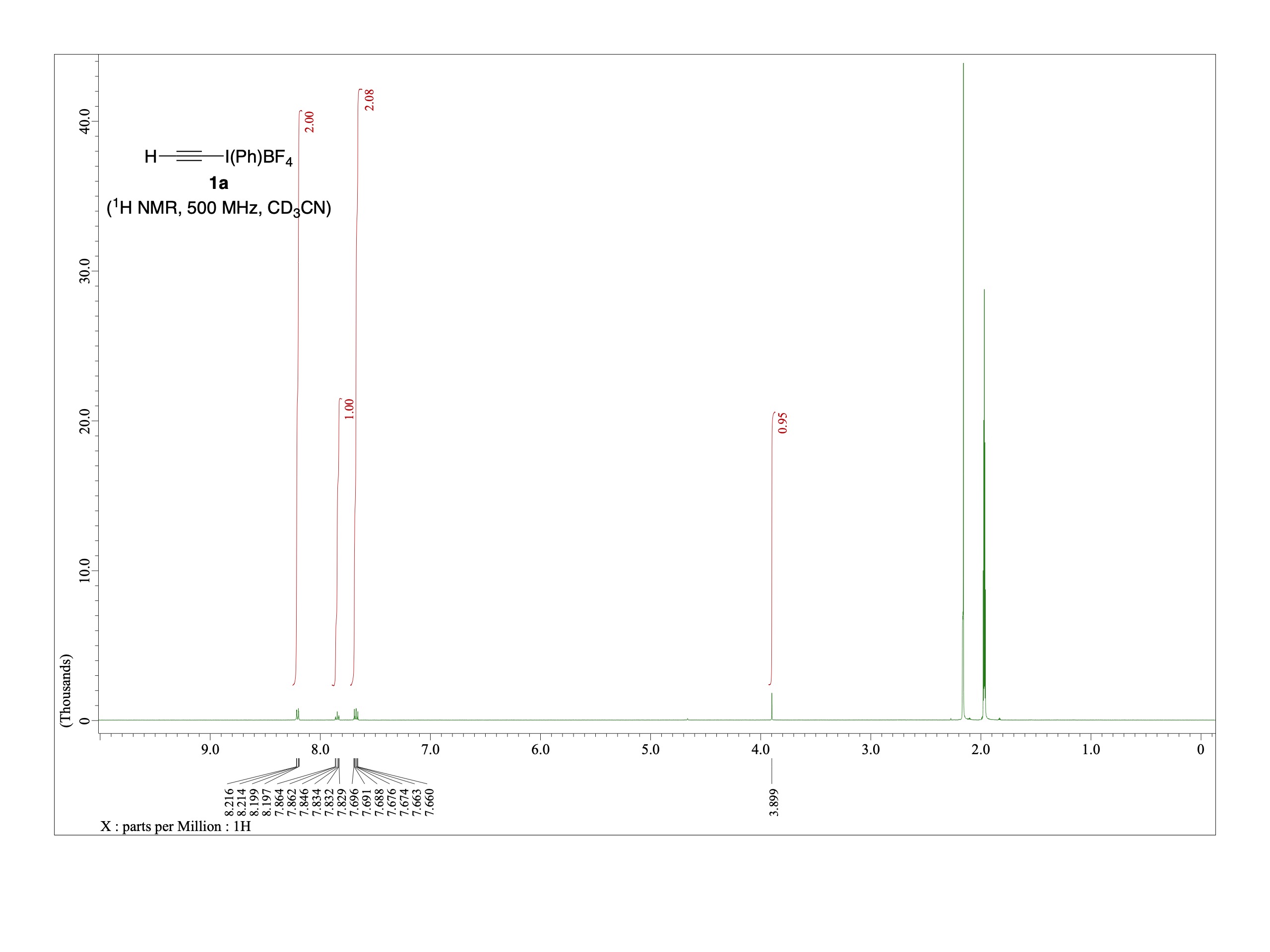

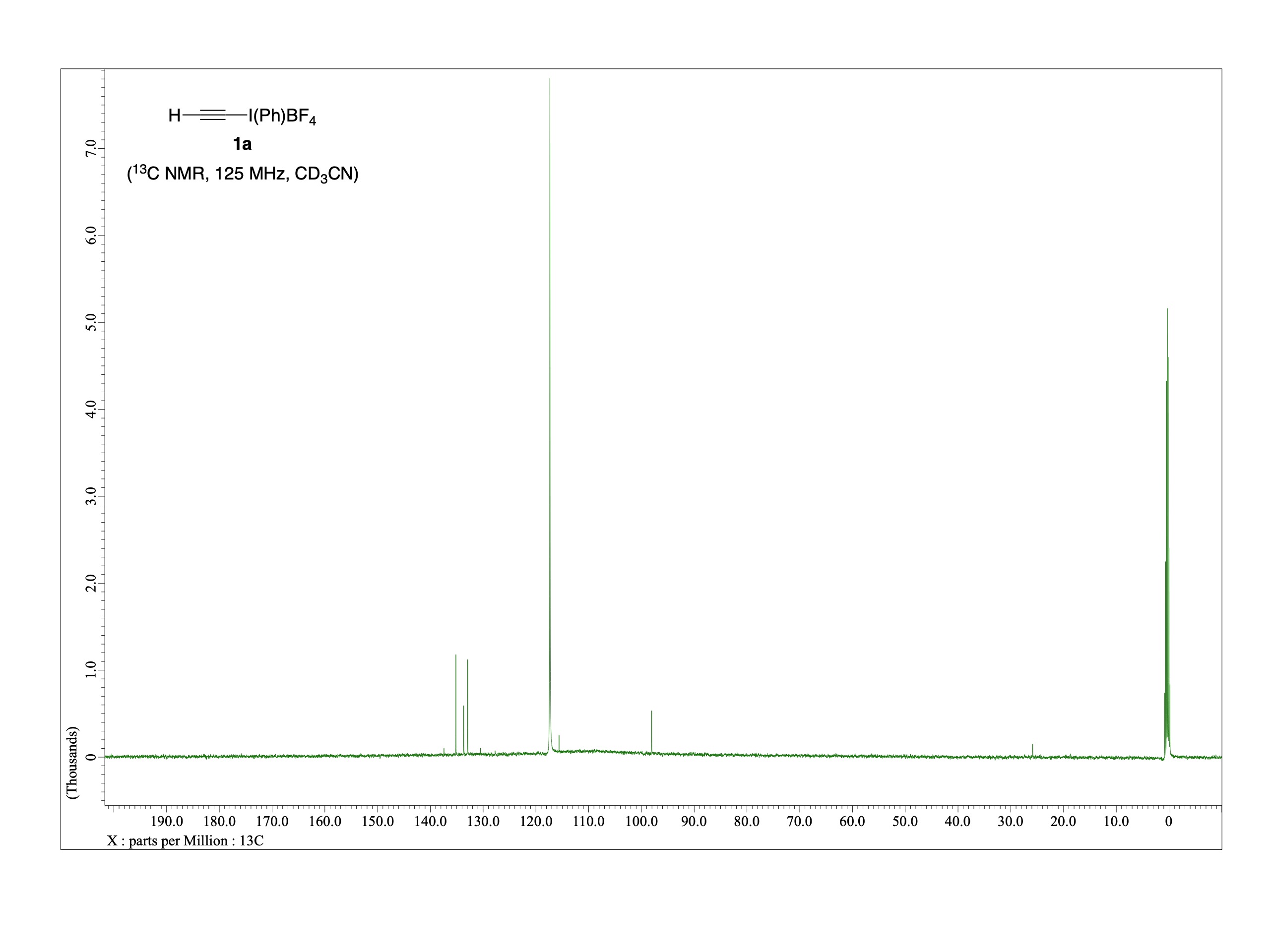


S4
